# Appropriate screening mammography method for patients with breast implants

**DOI:** 10.1038/s41598-023-28399-1

**Published:** 2023-02-01

**Authors:** Jihee Park, Eun Young Ko, Boo-Kyung Han, Eun Sook Ko, Ji Soo Choi, Haejung Kim

**Affiliations:** Department of Radiology, Center for Imaging Science, Samsung Medical Center, Sungkyunkwan University School of Medicine, 81 Irwon-Ro, Gangnam-Gu, Seoul, 06351 Korea

**Keywords:** Health care, Oncology

## Abstract

In this study, we aimed to evaluate the benefits and losses of mammography with and without implant displacement (ID) and propose an appropriate imaging protocol for the screening of breasts with implants. We evaluated mammograms of 162 breasts in 96 patients including 71 breasts with biopsy-proven cancers. Mammography of each breast included standard MLO and ID MLO images. We reviewed the mammograms using clinical image quality criteria, which consist of parameters that evaluate the proper positioning of the breast and the image resolution. Standard MLO images showed significantly higher scores for proper positioning but showed significantly lower scores for image resolution than the ID MLO images. Moreover, standard MLO images showed significantly higher kVp, mAs, and compressed breast thickness than the ID MLO images. The organ dose was also higher in the standard MLO images than in the ID MLO images, but the difference was not statistically significant. In mammography with proven cancer, ID MLO images showed significantly higher degree of cancer visibility than standard MLO images. For screening mammography in patients with breast implants, ID MLO view alone is sufficient for MLO projection with reducing the patient’s radiation dose without compromising the breast cancer detection capability, especially in dense breasts with subpectoral implants.

## Introduction

Screening mammography is the most widely used breast cancer screening tool and is the only imaging modality proven to significantly lower breast cancer mortality^1^. Guidelines for obtaining screening mammography images are relatively clear and widely followed. Two standard views for each breast, namely the craniocaudal (CC) and mediolateral oblique (MLO) views, are the most commonly used imaging techniques^2,3^. However, the imaging guidelines for screening mammography in patients with breast implants remain unclear^4^. Therefore, different sets of mammograms are usually acquired for screening patients with breast implants. Depending on the institution, standard mammography for screening may include four images without implants in both CC and MLO views, eight images with and without implants in both CC and MLO views, or six images consisting of four images with and without implants in MLO view and two images without implants in CC view.

There are reports that mammography with implants incurs a higher radiation dose than mammography without implants^5^. Current imaging protocols in many institutions include mammography with implants, as it is unclear whether they are more beneficial for cancer detection. Studies comparing the benefits and losses of mammography with and without implants with respect to and in relation to mammography image quality or performance for breast cancer detection are rare. Available published studies have only compared the radiation dose according to mammography techniques^5^. Even if mammography with implants has a higher radiation dose, it may still be of benefit if the images provide additional information that increases the ease of breast cancer detection. On the contrary, if there is little benefit for cancer detection than considering the higher radiation dose, the images should not be used for screening. To date, research data to support evidence-based decision-making is lacking.

The lack of guidance has led to wide variations in mammography practice for breasts with implants. As a result, heterogeneous examinations, with varying image quality and radiation doses, may be of concern^6^.

This study aimed to evaluate the benefits and losses of mammography with and without implant displacement and to propose an appropriate imaging protocol for screening mammography of breasts with implants.

## Methods

In this study, we defined standard images as mammograms with implants and implant displacement (ID) images as mammograms without implants. The institutional review board of Samsung Medical Center approved this retrospective study and waived the requirement for informed consent (IRB 2021-09-121). All methods were performed in accordance with the relevant guidelines and regulations.

### Subjects

We retrospectively evaluated mammography of 96 consecutive patients who had newly diagnosed breast cancers and breast implants between January 2018 and December 2019. Among the 192 breasts of 96 patients with implants, six breasts that were too small or the cancer was too large to displace the implant posteriorly, and those that failed to obtain an ID view were excluded. Mammograms of 24 breasts that did not provide data of digital imaging and communications in medicine (DICOM) header information on the radiation dose were also excluded. In total, 162 breasts of 96 patients (71 breasts with biopsy-proven cancers and 91 breasts without proven cancer) were included in our study. The mean age of the patients was 43.7 ± 6.8 years (range, 36.9–50.5 years) and most implants were subpectorally located.

### Mammogram acquisition

All mammography images were obtained using digital mammographic units, Selenia Dimensions (Hologic, Bedford, MA, USA) or Senographe 2000D (GE Medical Systems, Milwaukee, Wis, USA). Three images were obtained for all breasts: standard MLO, ID MLO, and ID CC views. ID MLO and ID CC views were obtained under the full automatic exposure control mode like the breasts without implants. To obtain the ID image, the implant was gently pushed back by hand and displaced posteriorly, while compression was applied to the breast tissues anterior to the implant^7^. For standard MLO view, mammography including implant was obtained under “implant mode” with automatic exposure control in GE mammography system. In the case with insufficient contrast of breast parenchyma with “implant mode”, we repeated the acquisition with manual setting, however it rarely happened. In Hologic mammography system, we obtained standard MLO view with manual setting of exposure considering the thickness and composition of compressed breast. If the obtained image including implant showed inappropriate contrast of breast parenchyma, “implant present” function was applied for the post-processing of acquired image to make better visualization of breast parenchyma.

The imaging process was performed by experienced breast mammographic technologists.

### Image analyses

We compared the two MLO images. Two radiologists retrospectively reviewed the standard MLO images and ID MLO images of the 162 breasts. The clinical image quality was assessed as per predetermined criteria in consensus. The criteria used for evaluating clinical image quality consist of parameters that evaluated proper positioning and parameters that evaluated image resolution. To assess proper positioning, the following parameters were evaluated: the inferior border of the pectoral muscle (PM) at the correct level, inframammary fold (IMF) clearly demonstrated, presence of sagging and skin folds, the nipple in profile or overlapping with breast tissues, visualization of the retromammary fat, breast centrally placed within the image, and full visualization of the whole breast. To assess image resolution, the following parameters were analyzed: appropriate compression (spread of the breast tissues to differentiate adipose tissues from fibroglandular tissues), no blurring, and appropriate contrast. Each characteristic was scored categorically as either present or absent, or as good, fair, or poor. We extracted the clinical imaging quality criteria and the score assigned to each criterion from the study by Sá dos Reis et al.^4^ and clinical image evaluation criteria of mammograms used by Korea Society of Breast Imaging^7^.

We also investigated the imaging conditions and technical parameters by obtaining the information from the DICOM headers, including kVp, mAs, compressed breast thickness, and organ dose.

For the 71 breasts with proven cancer, two radiologists assessed the mammograms for the degree of cancer visibility and categorized the parameter as nonvisible, detected but inconclusive, and considered malignant. Factors influencing the degree of cancer visibility were also assessed.

### Data analysis

We compared the clinical image quality, imaging conditions, and technical parameters of the standard MLO and ID MLO images. If there was a parameter showing a difference between the two images, the standardized mean difference (SMD) using the effect size was calculated to determine which characteristic differed most significantly between the two images. The effect size was described as small (≤ 0.2), medium (> 0.2 and ≤ 0.8), and large (> 0.8)^8^. We also evaluated the imaging conditions that were closely related to the most significantly different characteristics between the two images.

To analyze the clinical image quality criteria, McNemar’s test was used for categorical variables that were divided into two categories, and Bowker's symmetry test was used for variables that were divided into three categories^9,10^. The Wilcoxon signed-rank test was used for continuous variables. McNemar's test was also used to compare cancer visibility between the standard MLO and ID MLO views. Logistic regression was used to analyze the factors associated with the degree of cancer visibility. The *p*-value < 0.05 was considered statistically significant. Statistical analysis was performed using SAS version 9.4 (SAS Institute, Cary, NC) and R 4.0.4 (Vienna, Austria; http://www.R-project.org/).

## Results

**Figure 1:**
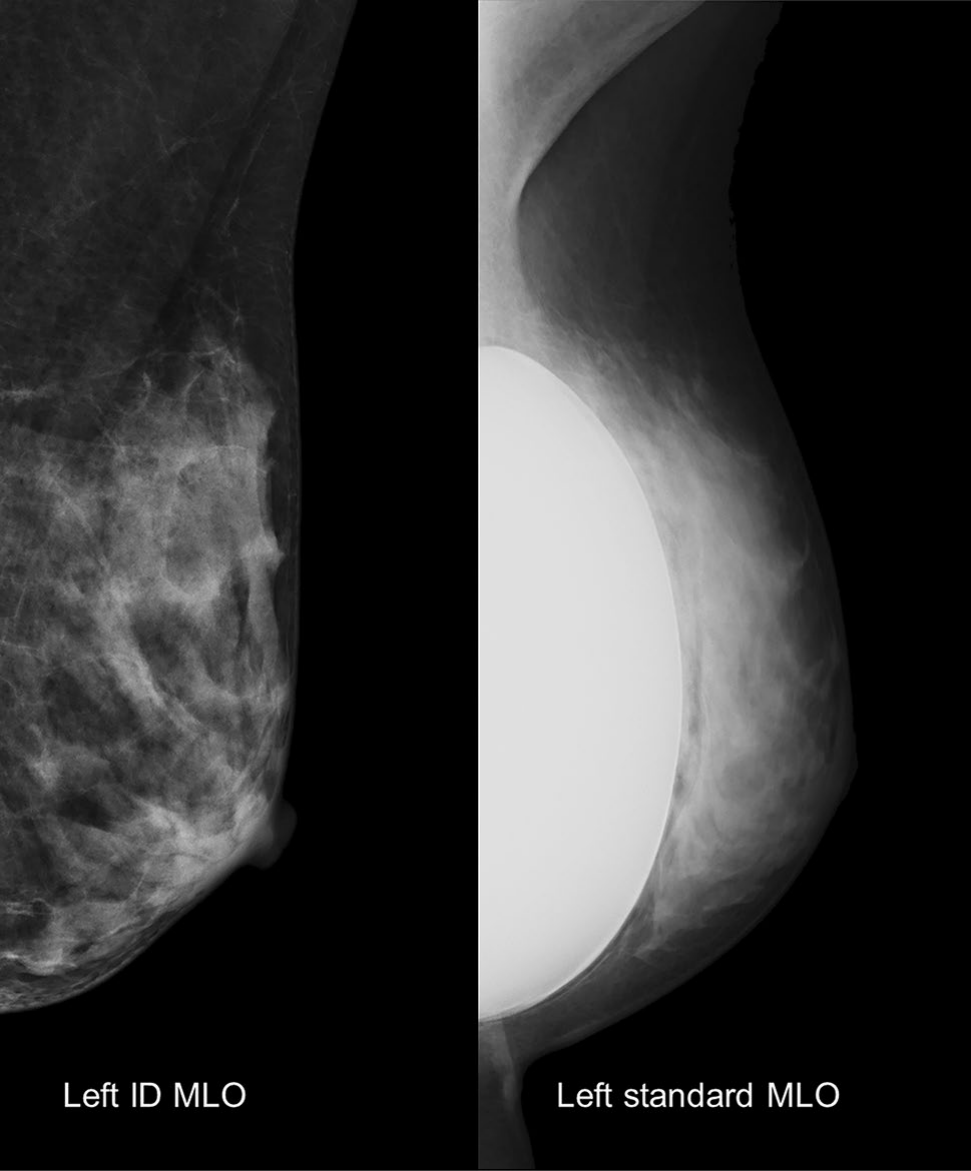
Screening mammography of a 36-year-old woman with subpectoral breast implants. The ID MLO image of the left breast shows insufficient visualization of the retromammary fat, insufficient margin of the whole breast, and IMF. The ID MLO view shows better compression and contrast. The standard MLO image shows more breast tissues, including a part of the retromammary fat and IMF, than the ID MLO image. However, the standard MLO view shows poor compression and contrast. *ID MLO* implant displaced mediolateral oblique, *IMF* inframammary fold.

Standard MLO images showed significantly higher scores in most parameters for proper positioning but showed significantly lower scores in parameters for image resolution than ID MLO images, except for blurring (Table 1, Fig. 1). Full visualization of the whole breast tissue, appropriate compression, appropriate contrast, and fully demonstrated IMF showed a significant difference between the two images (SMD > 0.8). The order of significance was the same as that described above.

**Table 1:** Comparing image quality between the standard and ID MLO views according to the clinical image quality scoring system (N = 162).

Clinical IQ criteria	Characteristics	Meaning	Score	ID MLO	Standard MLO	*p*-value	SMD
Positioning	Inferior border of PM	Above the level of the posterior nipple line	0	15 (9.3)	2 (1.2)	0.0003	0.366
		Down to the level of the posterior nipple line	3	147 (90.7)	160 (98.8)		
	Sagging	Clearly present	0	0	0	0.0348	0.176
		Partially present	3	14 (8.6)	7 (4.3)		
		Absent	5	148 (91.4)	155 (95.7)		
	IMF demonstration	Poor	0	162 (100.0)	120 (74.1)	< 0.0001	0.837
		Fair	3	0 (0.0)	25 (15.4)		
		Good	5	0 (0.0)	17 (10.5)		
	Skin folds	Clearly present	0	9 (5.6)	6 (3.7)	0.003	0.261
		Partially present	1	51 (31.5)	71 (43.8)		
		absent	2	102 (63.0)	85 (52.5)		
	Nipple in profile or overlapped with breast tissue	Nipple overlaps the breast completely	0	3 (1.9)	1 (0.6)	0.0186	0.263
		Nipple overlaps the breast partially	1	21 (13.0)	10 (6.2)		
		Nipple in profile	2	138 (85.2)	151 (93.2)		
	Visualization of retromammary fat	Poor	0	67 (41.4)	28 (17.3)	< 0.0001	0.682
		Fair	3	66 (40.7)	63 (38.9)		
		Good	5	29 (17.9)	71 (43.8)		
	Breast centrally placed	No	0	20 (12.3)	1 (0.6)	< 0.0001	0.49
		Yes	3	142 (87.7)	161 (99.4)		
	Full visualization of whole breast tissue	No	0	143 (88.3)	3 (1.9)9)	< 0.0001	3.503
		Yes	5	19 (11.7)	159 (98.1)		
Image resolution	Appropriate compression	Poor	0	2 (1.2)	107 (66.0)	< 0.0001	2.133
		Fair	3	70 (43.2)	46 (28.4)		
		Good	5	90 (55.6)	9 (5.6)		
	No blurring	Severe blurring	0	0	0	0.3173	0.111
		Mild to moderate blurring	3	0 (0.0)	1 (0.6)		
		No blurring	5	162 (100.0)	161 (99.4)		
	Appropriate contrast	Poor	0	2 (1.2)	97 (59.9)	< 0.0001	1.956
		Fair	3	58 (35.8)	51 (31.5)		
		Good	5	102 (63.0)	14 (8.6)		

*IQ* image quality, *ID* implant displaced, *MLO* mediolateral oblique, *PM* pectoralis muscle, *IMF* inframammary fold, *SMD* standardized mean difference.

Regarding the imaging conditions and technical parameters, standard MLO images showed significantly higher kVp (29.00 vs. 27.00) (*p* < 0.0001), mAs (76.00 vs. 99.00) (*p* < 0.0001), and thicker compressed breast thickness than ID MLO images (33.00 mm vs. 65.50 mm) (*p* < 0.0001). The organ dose was also slightly higher in the standard MLO images than in the ID MLO images, but the difference was not statistically significant (Table 2).

**Table 2:** Imaging conditions and technical parameters.

Characteristics	ID MLO (*N* = 162)	Standard MLO (*N* = 162)	*p*-value
Compressed breast thickness	33.00 (28.00, 39.00)	64.50 (57.25, 72.75)	< 0.0001
kVp	27.00 (26.00, 28.00)	29.00 (28.00, 29.00)	< 0.0001
Exposure (mAs)	76.00 (47.00, 98.75)	99.00 (63.00, 138.00)	< 0.0001
Organ dose (mGy)	0.95 (0.79, 1.11)	0.97 (0.75, 1.30)	0.0954

The value in parenthesis is range.

*ID* implant displaced, *MLO* mediolateral oblique.

**Figure 2:**
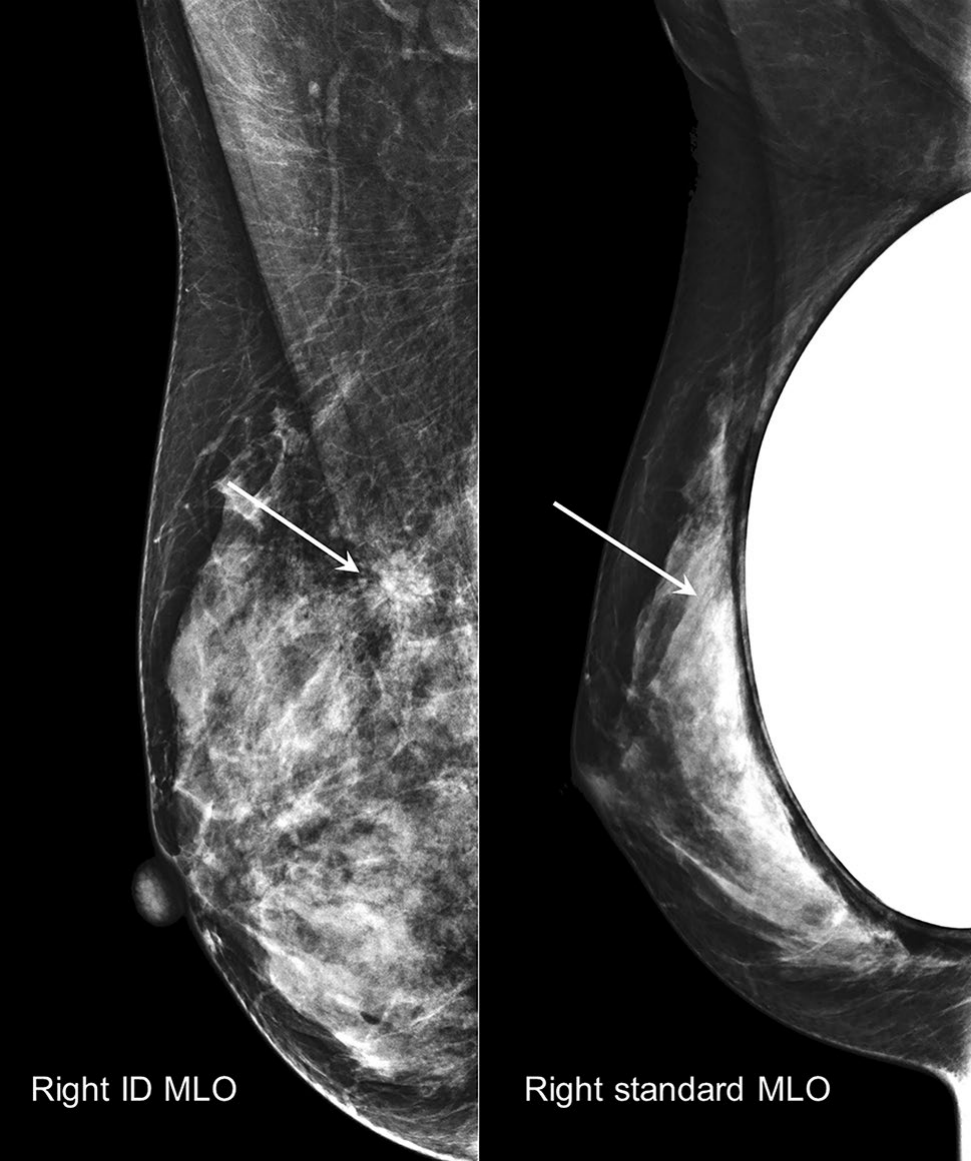
Screening mammography of a 36-year-old woman with subpectoral breast implants. The ID MLO image shows an irregular shape, spiculated, high density mass (arrow) in the right upper breast. Biopsy of the mass revealed invasive ductal carcinoma. On the standard MLO image, the cancer is not detectable due to tissue overlapping, with poorly spread and inappropriately compressed dense parenchyma. *ID MLO* implant displaced mediolateral oblique.

The clinical characteristics of the breasts and the histologic characteristics of proven breast cancers in 71 patients are listed in Table 3. Most patients had dense breasts (91.5%) and subpectoral type of silicone implants (93.0%). Mean invasive tumor size was 1.0 ± 1.0 cm and 87.3% was T-stage 0 or 1. Hormone receptor positive and Her2 negative subtype was predominant (83.1%). For the breasts with proven cancers, ID MLO images showed a significantly higher degree of cancer visibility than the standard MLO images (Table 4) (Fig. 2). None of the breast cancer not seen in ID MLO image was found in standard MLO image (n = 24). On the other hand, 35.1% (13/37) of breast cancers that were not seen in standard MLO view were detected in ID MLO view, and 24.3% (9/37) of them showed suspicious findings suggestive of breast cancer. Although the difference in the number of cases was too large to have statistical significance, the difference in cancer visibility between standard MLO and ID MLO view was greater in dense breasts than in fatty breasts (29 vs 41 in dense breasts, 5 vs 6 in fatty breasts), and slightly more in prepectoral than.retropectoral implants (3 vs 1 in prepectoral implants, 44 vs 33 in retropectoral implants). Table 5 shows the image features of 71 breast cancers and their visibility on the standard and ID MLO views. Mammography could detect 66.2% of breast cancers, and there was significant difference in overall cancer visibility between the two views (*p* < 0.0001). Most common findings on mammography was a high density mass with irregular shape and indistinct or speculated margin. Suspicious calcifications without definite mass was the second most common finding. However, difference in cancer visibility between the standard and ID MLO views did not vary depending on the type of mammographic feature (*p* = 0.115*).*

**Table 3:** Clinical and histological characteristics of 71 breasts with proven cancers (N = 71).

		No. (%)
Patients		
Age (years)		44 ± 7 (range, 32–63)
Sex		All female
Breast density	Fatty	6 (8.5)
	Dense	65 (91.5)
Implants		
Location	Subpectoral	66 (93.0)
	Prepectotal (subglandular)	5 (7.0)
Type	Silicone	71 (100.0)
	Saline	0
Cancers		
Site	Right (n = 32)	Left (n = 39)
	
T-stage	Tis	20 (28.2)
	T1	42 (59.2)
	T2	6 (8.5)
	T3	1 (1.4)
Size (cm)	Invasive cancer	1.0 ± 1.0 (range, 0–5.5)
	Invasive + In situ carcinoma	2.4 ± 1.9 (0.015–8)
Subtype	ER ( +), Her2 ( −)	59 (83.1)
	ER ( +), Her2 ( +)	4 (5.6)
	ER ( −), Her2 ( +)	2 (2.8)
	ER ( −), Her2 ( −)	6 (8.5)

*SA* subareolar, *W* whole breast.

**Table 4:** Comparing cancer visibility between Standard and ID MLO views (N = 71).

Characteristics	Score	ID MLO	Standard MLO			*p*-value
The degree of cancer visibility	0	24 (14.8)	37 (22.8)	Score in ID MLO		< 0.0001
				0	24	
				1	4	
				2	9	
	1	9 (5.6)	14 (8.6)	1	5	
				2	9	
	2	38 (23.5)	20 (12.3)	2	20	

The value in parenthesis is a percentage.

*ID* implant displaced, *MLO* mediolateral oblique.

Score 0 = nonvisible, 1 = detected but inconclusive, 2 = considered malignant.

**Table 5:** Mammographic findings of proven breast cancer and the visibility on Standard and ID MLO views (N = 71).

Finding		No. (%)	ID MLO	Standard MLO	*p*-value
Positive		47 (66.2)	47	34	< 0.0001
Mass		26 (36.6)	26	17	0.115
Shape	Round or oval	5			
	Irregular	21			
Margin	Circumscribed	0			
	Indistinct	14			
	Spiculated	12			
Density	Iso	1			
	High	25			
With calcifications		8	8	5	
Focal asymmetry		4 (5.6)	4	3	
Calcifications only		17 (23.9)	17	14	
Shape	Amorphous	3			
	Coarse heterogeneous	3			
	Fine pleomorphic/linear	11			
Distribution	Segmental	6			
	Grouped	7			
	Regional	2			
	Diffuse	2			
Negative		24 (33.8)			

*ID* implant displaced, *MLO* mediolateral oblique.

Appropriate compression and contrast of ID MLO images enabled better cancer visibility by approximately 3.0-fold when compared with the standard MLO images. However, these differences were not statistically significant. The only factor that showed a statistically significant association with the difference in cancer visibility was the resolution score, which consisted of the sum of the scores for appropriate compression, appropriate contrast, and blurring (*p* = 0.0197) (Table 6).

**Table 6:** Factors associated with cancer visibility.

Characteristics	Odds ratio	95% CI		*p*-value
Inferior border of PM				
*0	Ref			
**1	0.297	0.011	8.146	0.4727
Sagging				
0	Ref			
1	1.532	0.256	9.161	0.6404
IMF demonstration				
0	Ref			
1	0.661	0.188	2.321	0.5181
Skin folds				
0	Ref			
1	1.397	0.372	5.242	0.6203
Nipple in profile or transected by skin				
0	Ref			
1	0.565	0.062	5.185	0.6134
Visualization of retromammary fat				
0	Ref			
1	1.337	0.412	4.341	0.6288
Breast centrally placed				
0	Ref			
1	0.821	0.154	4.369	0.8176
Full visualization of whole breast tissue				
0	Ref			
1	0.447	0.11	1.81	0.259
Appropriate compression				
0	Ref			
1	3.022	0.351	26	0.3139
No blurring				
0	Ref			
1	9.301	0.096	902.307	0.3393
Appropriate contrast				
0	Ref			
1	3.022	0.351	26	0.3139
Sum of positioning score	1.025	0.888	1.183	0.7326
Sum of resolution score	1.256	1.037	1.521	0.0197
Total score (positioning and resolution)	1.104	0.937	1.3	0.2381
Compressed breast thickness	1.042	0.993	1.093	0.0908
kVp	1.227	0.914	1.648	0.1731
Exposure (mAs)	0.996	0.979	1.013	0.6341
Organ dose_MLO (mGy)	1.506	0.367	6.188	0.5698

*CI* confidence interval, *PM* pectoralis muscle, *IMF* inframammary fold, *MLO* mediolateral oblique.

*When both ID MLO and standard MLO identified cancer or did not detect cancer.

**When only one of the ID MLO and standard MLO views detected cancer.

## Discussion

In patients with proven breast cancers, standard MLO images showed a lower degree of cancer visibility and a higher radiation dose than ID MLO images. Although the standard MLO images demonstrated higher scores in the positioning criteria, these did not lead to additional information or greater advantages for the detection or diagnosis of breast cancer. Instead, only the radiation exposure was increased.

To obtain a standard MLO image, with the breast implant included within the image, the breast tissue located anterior to the implant must be fully included within the image. In other words, the IMF, retromammary fat, and the margin of the peripheral breast tissue should be included in the image. However, in the standard MLO image (with implant), the included breast parenchyma is pushed from behind by the implant and pressed without spreading properly. In contrast, while obtaining the ID MLO image of the breast, a gentle hand pushing maneuver displaces the implant posteriorly, and the peripheral portion of the breast can also be pushed back together. Thus, in the ID MLO view (without implant), it can be difficult to fully include the peripheral portion of the breast. The breast should be pulled inside the field and the implant should be pushed outside the field. Then the included breast parenchyma can spread well and be compressed without obstacles. Importantly, the ID MLO views showed significantly higher scores in terms of image resolution. These results support that image resolution, such as appropriate compression, contrast, and absence of blurring, are more important for cancer visibility than proper positioning in breasts with implants.

The criteria for proper positioning account for the majority of the score for the clinical image evaluation of mammograms. Correct positioning is an essential prerequisite for obtaining appropriate images in patients without implants using mammography. In general, an image that did not score well in proper positioning also did not have a high resolution score. However, this also applies to the absence of implants. Our study showed that ID MLO images received lower positioning scores but higher scores in resolution and cancer detection rates, suggesting that in patients with implants, it is inevitable that a little tissue of the posterior aspect of the breast is cut off from the image due to the implant being pushed back. However, this did not necessarily equate to poor clinical imaging quality.

In our institution, mammography for breast with implants includes standard MLO images in addition to four images in the ID MLO and ID CC views, because we are concerned that small breast cancer lesions in the peripheral parts of the breast may be excluded when obtaining the ID MLO view. However, even if there is a breast cancer lesion in the peripheral parts of the breast that may be more clearly included via standard MLO images, the compression of the breast tissues in standard MLO images is not sufficient. This is likely to result in the breast tissues overlapping with other tissues, thus hiding any cancer lesions at the periphery. In this study, there was no case in which an ID MLO view detected less breast cancer than the standard MLO view because a small peripheral part of the breast tissues was less visible. Based on our results, we suggest that for routine screening mammography, trying to inclusion of the IMF or retromammary fat while obtaining an ID MLO view of the breast would be preferable to adding standard MLO images.

Eklund et al.^11^ showed that a modified compression mammogram improved imaging in augmented breasts. However, they only stated that the modified compression mammogram should be added, and did not mention whether the standard images without ID should be maintained or whether the modified compression mammogram alone is sufficient. Reis et al.^4^ studied the image quality of mammograms performed in patients with breast implants. However, they did not compare mammography with and without implants, but only analyzed the image quality of mammograms for breasts with implants from multiple centers, which were performed with various projections and techniques. The authors suggested that the current criteria for evaluating the image quality of mammograms were not fully applicable to patients with implants. As there has been no other study that compared standard MLO views and ID MLO views in the same patients thus far, our results are meaningful and informative.

Reducing the radiation dose of screening tools such as mammography is recognized as increasingly important^5,12–15^. Due to the development of mammographic equipment and technology, even if the breast thickness increased significantly in the standard MLO view including implant, the radiation dose was not significantly increased compared with ID view (0.97 mGy vs 0.95 mGy, *p* = 0.0954). Although the radiation dose of each view was not high, the total dose received when the patient took both views was higher than the achievable mean dose for the screening (1.1 ± 0.5 mGy) and some of them exceeded the acceptable dose level of 2.00 mGy^16^. This suggests that obtaining both ID MLO and standard MLO images in breast cancer screening may lead to increase radiation dose without any particular benefits in breast cancer detection.

Sometimes, mammography is performed to evaluate implant rupture. In these cases, a view including the implant is essential. However, in general, it is more accurate to evaluate implant rupture using MRI or breast ultrasonography, rather than mammography^17,18^. Most screening mammography is not designed to evaluate the condition of the implant but to detect breast cancer. Hence, routinely performing standard MLO view of mammography in breast cancer screening to detect asymptomatic rupture of implant is not recommended^18^.

Our study had some limitations. First, we compared the image quality of standard MLO and ID MLO images using clinical image quality criteria and scores that were used to evaluate the quality of screening mammography. These criteria are not fully applicable to mammography with implants. However, these criteria were used as we did not have other objective criteria for the evaluation of mammograms with implants. Second, this study compared two different methods of mammography acquisition for breast with implants, but only in the MLO view. In most medical centers, screening mammography imaging protocols in patients with breast implants usually include both ID MLO and ID CC views, or additional two MLO views without ID rather than the CC view. Third, we did not compare the results between the two different mammographic units we had, nor analyze the data separately according to the mammographic unit. There is a possibility that the relative dose between ID and standard MLO views can be different according to the mammographic system. Finally, this study is a single-center study and the population is predominantly women with dense breasts and subpectoral silicon implants. In other populations, the results may not be the same. In our institution, we performed mammography in the same way regardless of breast size, the type or volume of implant. Additional studies from multiple centers are required. Applying different acquisition methods on the type or size of the implant may obtain different results.

In conclusion, for screening mammography in patients with breast implants, acquiring both standard MLO and ID MLO view do not have benefit compared with ID MLO view alone. For MLO projection, ID MLO view is sufficient and is expected to reduce the patient’s radiation dose, without reducing the capability of breast cancer detection, especially in dense breasts with subpectoral implants.

## Data Availability

The datasets used and/or analyzed during the current study available from the corresponding author on reasonable request.
